# Extract from a Report of the Standing Committee on Surgery, Read before the Kentucky State Medical Society Oct. 1853

**Published:** 1854-01

**Authors:** Joshua Flint

**Affiliations:** Professor


From the Medical Examiner.

*Extract from a Report of the Standing Committee on Surgery, read before the Kentucky State Medical Society, Oct.* 1853. By Joshua Flint, Professor

The passion for operating, always sufficiently conspicuous in miscellaneous practice, has displayed itself, at different times, with appalling recklessness, in the conduct of specialities, and gathered its bloody laurels under favor of a present infatuation or panic respecting a particular form of disease. The *circulatores* of the middle ages, and the *emasculatores* of the eighteenth century, present remarkable illustrations of surgery run mad, in times past, while the *myotomists*, the *ovariotomists*, and the *womb-burners* of our own day, may fairly contend for the distinction of furnishing a modern parallel.

A tempting field for indulging the 'rage for cruel and bloody operations,' which Dr. Lee says 'has spread far and wide in England, and threatens to pervert and corrupt the sound and fundamental doctrines of British practice,' is found in the treatment of morbid growths and malignant degenerations presenting themselves in the form of tumors, on the surface or in the cavities of the body. In the management of these diseases, a class of questions arises, the determination of which often involves the character of surgery in general, as well as that of the practitioner immediately interested, and exhibits, in signal contrast, the sound, conservative surgery of science, and that flippant, reckless counterfeit of it, that rests in artisanship.

In the midst of these perplexing questions of diagnosis and treatment, with which such cases often confound the most competent practitioner, the true surgeon pauses, watches, consults, palliates, and perhaps cures—the hero of the scalpel cuts the gordian knot, and with it, perhaps, the life-thread of the patient.

No grave operation should be undertaken upon a mere vague *hope* of success. The prudent and consciencious surgeon will act upon nothing short of a reasonable probability of definity and sufficient advantage to the patient, at a compensation for the pain and injury inflicted on him.

But the patient will certainly die if something be not done. With all deference, my enterprising friend, you must not say that so positively, and even if it were undoubtedly true, I must be allowed to add, it is none of your business. If you have any mode or means of relief for the sufferer, proceed, at once, to employ it, under the general responsibility of the healing-art—responsibilities the force of which can neither be augmented nor diminished by your conjectures about the duration of the patient's life. The Supreme author and arbiter of life has reserved all knowledge, on that subject, to himself; but has given a solemn lesson of its sacredness in his sight, in that august command—addressed as well to the surgeon as to the highwayman—"Thou shalt not kill."

